# The architecture of *network collective intelligence*: correlations between social network structure, spatial layout and prestige outcomes in an office

**DOI:** 10.1098/rstb.2017.0238

**Published:** 2018-07-02

**Authors:** Felichism Kabo

**Affiliations:** Institute for Social Research, University of Michigan, Ann Arbor, MI, USA

**Keywords:** social networks, collective intelligence, physical space, prestige, individual level, team level

## Abstract

A social network represents interactions and knowledge that transcend the intelligence of any of its individual members. In this study, I examine the correlations between this *network collective intelligence*, spatial layout, and prestige or status outcomes at the individual and team levels in an organization. I propose that spatially influenced social cognition shapes which individuals become members of prestigious teams in organizations, and the prestige perception of teams by others in the organization. Prestige is a pathway to social rank, influence and upward mobility for individuals in organizations. For groups, perceived prestige of work teams is related to how team members identify with the group and with their collaborative behaviours. Prestige enhances a team's survivability and its access to resources. At the individual level, I ran two-stage Heckman sample selection models to examine the correlation between social network position and the number of prestigious projects a person is a member of, contingent on the association between physical space and social ties and networks. At the team level, I used linear regressions to examine the relationship among network structure, spatial proximity and the perceived prestige or innovativeness of a project team. In line with my hypotheses, for individuals there is a significant correlation between physical space and social networks, and contingent on that, between social network positions and the number of prestigious projects that a person is a member of. Also in accordance with my hypotheses, for teams there is a significant correlation between network structure and spatial proximity, and perceived prestige. While cross-sectional, the study findings illustrate the importance of considering the spatial domain in examinations of how network collective intelligence is related to organizational outcomes at the individual and team levels.

This article is part of the theme issue ‘Interdisciplinary approaches for uncovering the impacts of architecture on collective behaviour’.

## 1. Introduction

Interpersonal interaction and collaboration are key ingredients for individual and team thriving in social groupings including organizations. This is not a recent phenomenon. In fact, there are remarkable similarities between groups as diverse as hunter–gatherer bands [1] and work teams and organizations [2] with respect to dependence on their members' ability to collaboratively identify and exploit resources critical to individual and group outcomes. Cognition plays a major role in this process. Importantly, cognition is not merely confined to social knowledge, but also includes knowledge of physical space. More precisely, spatial cognition shapes social or socially shared cognition, which is, in turn, constituted by interactions among individuals [3,4]. Understanding socially shared cognition requires an analytic focus on the group's pattern of social interactions, its social network. The social network represents interactions and knowledge that transcend the intelligence of any of its individual members [5]. In this study, I examine the correlations between this *network collective intelligence*, spatial layout, and prestige or status outcomes at the individual and team levels in an organization.

I propose that spatially influenced social cognition shapes which individuals become members of teams in organizations, and how teams are perceived by others in the organization. I define the organization as a group of groups, where individuals and groups have to compete for the scarce resources that are critical to success and survival [6]. The pursuit of status and prestige are part of this competition and are universal to all social groupings including status hierarchies such as organizations [7,8]. *Expectations state theory* predicts the emergence of status hierarchies under conditions of task orientation (individuals motivated to solve a problem) and collective orientation (consideration of others' contributions in task completion) [9]. Prestige represents a strategy or pathway to navigating these hierarchies and to status acquisition [10]. For individuals within groups, the concept of prestige captures the cognitions, behaviours and emotions aimed at status attainment through displays of skill and knowledge [10–12]. Prestige is a pathway to social rank, influence and upward mobility for individuals in organizations [12,13]. For groups, research has shown that the perceived prestige of work teams is related to how team members identify with the group and with their collaborative behaviours [14,15]. Prestige enhances a team's survivability and its access to resources. For example, a project team that is perceived to be prestigious and innovative is likely to have higher levels of external contact and communication with other individuals and groups in the organization [16]. This communication is central to how team members seek information, resources and support within the organization [17].

**Figure 1.**
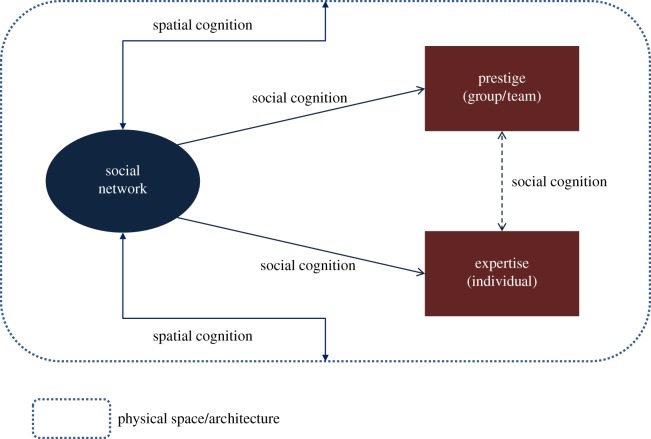
Relationship between social networks, space and (*a*) identification of expertise in individuals and (*b*) perception of a project team as prestigious and innovative. These relationships are actualized via social cognition, which is itself influenced by spatial cognition of the physical space or architecture. (Online version in colour.)

Interactions within social networks facilitate social cognition about which individuals have what ability or expertise. The *theory of transactive memory* proposes that, by communicating and interacting in networks, group members are able to identify and leverage the skills and expertise of others in the group [18]. Socially shared cognition, the collaboration among group members as they collectively encode, interpret and recall information [19], is thus a function of the social network. By interacting, individuals identify expertise in others that can be leveraged in projects critical to the organization's survival. But once these projects acquire the human resources with the requisite skill sets needed to ensure their success, then this feeds into the social cognition that informs the perceived prestige of project teams in the organization. This is the process captured by the framework in figure 1. I also examine whether networks are correlated with the composition of teams that are perceived as prestigious and innovative by organizational insiders. I hypothesize that an individual's place in the organization's social network structure plays a role in their membership in teams with high prestige, which has positive benefits for an individual's sense of self and career [14,15]. Where as prior research focused on the link from perceived prestige to group performance, in this study, I examine the association between: (i) network centrality, spatial layout and the frequency with which an individual is a member of prestigious project teams and (ii) the association between the perceived prestige of a project team, and the group's network structure and collective spatial proximity. Note that while the framework suggests that physical space impacts prestige through its effects on social networks, it also allows for the association between social networks and individuals' spatial assignment and location.

A social network is the pattern of relations and interactions among the individuals in a group. Moreover, the network has resources—which are sometimes referred to as *social capital*—that individuals can access and use [20,21]. Network position leads to individual differences in social capital. However, the social network is also a *collective intelligence* where the group whole is greater than the sum of individual intelligences [22]. My discussion of spatial and social cognition notwithstanding, I conceptualize ‘collective intelligence’ in network rather than in cognitive terms. In fact, I argue that the connectedness and immersion of humans in social networks means that collective decisions can be understood even if they are not declared explicitly [5]. However, I also acknowledge that physical space plays a significant role in the creation, maintenance and death of social ties and networks [23–25]. A key mechanism behind the influence of space on social networks is the structuring of face-to-face interpersonal encounters and interactions. Face-to-face (as opposed to electronically mediated) communication and encounters rely on the latent potential of physical space to structure potential encounters, which is contingent on the co-location of individuals.

The spatial layout can hinder or enhance the formation of social ties and networks, the operative mechanism being the impact of spatial cognition on social or socially shared cognition. This type of spatially influenced social cognition shapes the group networks that are formed via interactions between individuals. Moreover, individuals and groups experience the networks and physical space as structures of constraints and opportunities that are associated with individual and group outcomes. My thesis is that conceptualizing how social networks operate or function in physical space allows better understanding of the effects of network structure on individual and group outcomes. Next, I will review the research on spatial cognition, and socially shared cognition and social networks.

### (a) Spatial cognition

An individual's knowledge of the world is first experienced through the body and the spatial world that the person inhabits and interacts with [26]. Cognition, perception and action are shaped by the movement and interaction of our bodies in the physical environment [27]. *Spatial cognition* refers to various aspects of cognitive processing such as spatial: perception, memory and navigation or wayfinding [28]. Spatial cognition operates at multiple scales ranging from the space: of the body, around the body, of navigation and of external representations [29]. The space of the body has both perceptual (external and internal sensations) and behavioural (what the body does) dimensions [29]. The space around the body is the space of what we can immediately affect, as well as what immediately affects us [29]. The space of navigation or potential travel is the space of how we move around in buildings, cities and natural landscapes. Space is stitched together experientially, perceptually, from actual navigation, or cognitively via maps or descriptions [29]. Lastly, the space of external representations is created by people to aid cognition, such as the use of maps or architectural drawings to represent actual spaces [29]. I focus on the impacts of networks on individuals and teams confined to the spatial layout of an office floor, privileging the space of navigation and space around the body.

With respect to my thesis, spatial cognition is also inextricably intertwined with, and influences socially shared cognition. Not only do group members need to know who knows what and who is good at what, but they also need to know who is where. Spatially influenced social cognition confers advantages to individuals; those with more of it better know who is where in the organization's physical space.

### (b) Socially shared cognition and social networks

*Socially shared cognition* refers to the process by which individuals in groups collaboratively acquire, encode, store, interpret, recall, transmit and use information in the creation of a collective intellectual outcome [19,30]. The interactions among the individuals, the social network, create a structure that impacts the patterns of communication and distribution of information among the group members. This has appreciable impacts on the group's intellectual potential or collective intelligence. Analysing interactions is key to understanding social cognition [4]. Therefore, understanding the group's social network should yield insights into its collective intelligence. By their actions and interactions, individuals create the interconnections that structure the group's network.

Social network structure places some individuals in more or less advantageous positions compared with others. Individual network position is correlated with access to and potential for exploiting *social capital*, or network resources [31], and the prominence or visibility of the node in the network. In a knowledge-based workplace, the organizational network typically comprises individuals and teams in a multi-team membership web of relations [32]. Therefore, in addition to analysing the associations between individual prominence in the network and prestigious project membership, I also assess the relationship between network structure and the prestige perception of the project team at the group level. As all individuals in this study are simultaneously members of multiple prestigious and non-prestigious projects, my analysis plan enables me to identify whether network structure is associated with: (i) prestigious team membership for individuals and (ii) perception as ‘prestigious’ for project teams. With respect to my thesis, individual actions and interpersonal interactions result in a network that, in turn, becomes a source of constraints and opportunities for individuals and groups. Understanding the social network thus simultaneously reveals insights on how network structure shapes individual- and team-level outcomes in organizations [5].

## 2. Material and methods

### (a) Study sample and data description

**Figure 2.**
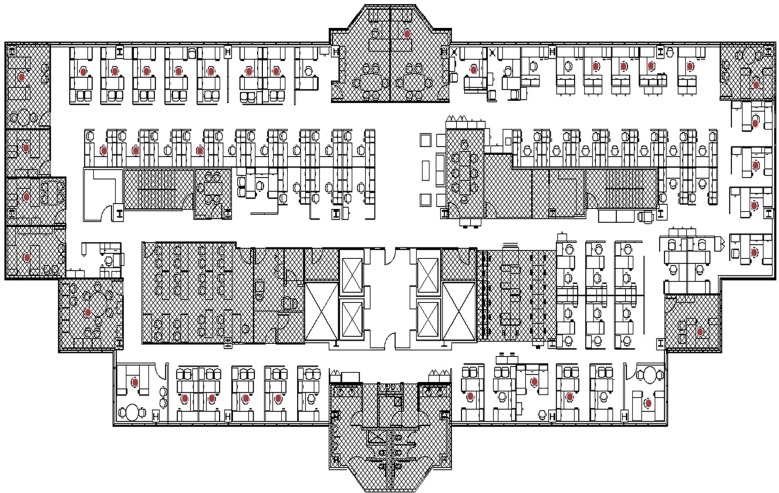
Floor plan for the study research site. The locations of the individuals in the study sample are indicated using the darkened circles/work chairs. One individual was frequently away at other sites belonging to the parent firm and would use any available workspace when on-site depending on meeting or work schedules. Therefore, this individual is not mapped on the floor plan. (Online version in colour.)

I administered an online sociometric survey in 2009 to a sample of 37 scientists and engineers who were employees of BRX, a unit responsible for environmental policy and compliance at a large manufacturing firm headquartered in the Midwest of the USA. The survey enabled me to collect data on multiple social networks including interaction, advice-seeking and advice-giving. In addition, I procured internal data on (i) sample attributes, e.g. gender, education and hierarchical or job status, (ii) AutoCAD^®^ drawing interchange (DXF) files of the workplace floor plan (figure 2), and (iii) information on the project team that individuals were affiliated with (*N* = 30) including an evaluation by organizational insiders on whether the project is prestigious and innovative. The organization was characterized by multiple team membership for individual employees. Excluding the director, who was nominally a member of all project teams, individuals at the organization were involved in a range of 0–21 project teams (mean = 6.56, median = 5, s.d. = 5.51). The project teams had a range of 4–15 members (mean = 8.8, median = 8, s.d. = 2.83).

### (b) Description of variables and order of analysis

#### (i) Individual level

The perceived prestige of a team augments group identification, which is in turn correlated with collaboration and interaction among team members [14,15]. The thrust of this study is to analyse whether there is a relationship between an individual's position in the network structure, spatial layout and the number of prestigious and innovative project teams that the individual is part of. I created the count dependent variable ‘number of prestigious/innovative projects’ that captured the number of prestigious project teams that the individual was a member of.

I used the interaction network item from the organizational sociometric survey to generate the social network measures of *degree* (related to the number of other individuals in the network that the person is directly connected to) and *betweenness* (how often a person is on the shortest paths between other pairs of individuals). Specifically, respondents were asked ‘About HOW OFTEN do you have discussions with this person to get your work done?’ The response options were: (i) 1 = monthly or less, (ii) 2 = several times a month, (iii) 3 = several times a week, (iv) 4 = daily and (v) 5 = several times a day. The degree measure indicates individuals that are prominent locally (immediate network neighbourhood). I binarized the degree measure at its median value to create the dependent variable ‘degree’ that was the outcome in the probit selection equation of the Heckman model (more details in the next section). The betweenness measure identifies individuals that are prominent globally (across the entire network). I used the betweenness measure to create the independent variable ‘betweenness’.

The spatial layout measure *integration* (the average depth of a space relative to all other spaces in the system) enables us to capture the ease with which a person can physically access other individuals at the workplace. The measure is grounded in ‘space syntax’, a theoretical perspective and set of methods that allow the conceptualization and analysis of physical space in network terms [33,34]. The measure was generated from a spatial network of the workplace created using the DXF files and Depthmap^®^ software. Spaces in this type of network are connected if they are adjacent and directly accessible from each other, i.e. it is physically possible to get from one space to the other. I used the integration measure to create the independent variable ‘integration’. Lastly, I created the control variables ‘gender’ (0 = female, 1 = male), ‘manager’ (0 = staff/non-manager, 1 = manager) and ‘graduate degree’ (0 = Bachelor's or lower, 1 = Master's or higher). Table 1 summarizes the individual-level variables.

**Table 1.** Summary statistics for individual-level variables.

variable	*n*	mean	s.d.	min.	max.
number of prestigious/innovative projects	37	1.3514	1.6024	0	6
degree	34	0.4412	0.5040	0	1
betweenness	34	1.1748	3.3622	0	15.226
integration	37	0.4111	0.0491	0.36	0.57
gender	37	0.5676	0.5022	0	1
graduate degree	37	0.5946	0.4977	0	1
manager	37	0.2432	0.4350	0	1

#### (ii) Group level

I worked with management to evaluate whether the organization's project teams (*N* = 30) were perceived to be prestigious and innovative (*n* = 6), or not (*n* = 24). My informants considered both external (for example, media or press coverage) and internal (for example, how they thought the project was perceived by executives from the parent firm) factors in evaluating whether a project was prestigious and innovative. I also wanted to control for the fact that project teams may vary with respect to the resources that are available, including human capital. Research has shown that while larger project teams have greater human resource capital, thereby enabling them to complete some tasks faster, there are also significant drawbacks to increasing a team's size [35]. Other work suggests that larger teams are more likely to reflect political and bureaucratic interests (such as a maximal head count to ensure representation of all stakeholders) rather than efficiency concerns [36]. I created the group-level dependent variable ‘size-weighted project prestige/innovativeness' as follows:2.1
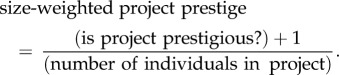
This variable controls for the possibility that resources, including human capital, might be shared unequally across teams and in favour of larger projects, positively skewing these teams' collective intelligence, and thus making it more likely that they are perceived as prestigious and innovative.

I used project team memberships to compute the group-level network variables. All 37 individuals in the sample had multi-team project memberships. Using the organizational interaction network, I extracted a subgraph for each project team. For each subgraph or team interaction network (*N* = 30), the independent variable ‘interaction network, in-degree centralization’ was generated by computing the *centralization* measures for the team interaction networks. Centralization captures the extent to which power and other positional advantages are distributed unequally in the network. The most unequal network has a star shape with the nodes on the periphery only having direct connections to the node in the centre. Network centralization informs on the variability of the distribution of positional advantages in a network relative to a star network of the same size (number of nodes or individuals in the present case). For the purposes of this study, centralization indicates the extent to which interactions among team members are concentrated in only a few of them as opposed to being distributed equally across the team [37].

Finally, I computed the *average weighted degree* of the spatial distances which is the average of the sum of weights of the edges, or in this case the distances among individuals in the team. This independent variable was created by generating a network from the matrix of distances among project members. In the spatial matrix, the edge's weight is the metric distance between individuals in a dyad or pair. Therefore the ‘spatial distance, average weighted degree’ is a function of the number of other individuals that person is connected to, and the distances from the focal individual to the other individuals. The measure thus indicates the overall spatial proximity within a team, while accounting for the team's size.

A key difference between edges in the interaction network and spatial distance matrix is that the former is directed (ties are directional, they can go *out* from the individual or *in* to the individual) while the latter are undirected (ties are bidirectional, or they simultaneously go to and from the individual). For the interaction networks, I computed ‘interaction network, in-degree centralization’ using *in-degree* or incoming ties as individuals that receive many ties are seen as prominent or high in prestige [38]. Table 2 is a summary of the group-level variables.

**Table 2.** Summary statistics for group-level variables.

variable	*n*	mean	s.d.	min.	max.
size-weighted project prestige/innovativeness	30	0.1532	0.0785	0.067	0.400
interaction network, in-degree centralization	30	0.0414	0.0103	0.030	0.085
spatial distance, average weighted degree	30	857.3571	323.2310	294.667	1469.989

### (c) Statistical analyses

#### (i) Individual level

I tested whether the multiple team membership of individuals violated the assumptions of independence in the data sufficiently to merit a multilevel modelling approach (with teams as a nesting variable) over a single-level model with no team effects. I fitted multilevel mixed effects linear and Poisson models with ‘number of prestigious/innovative projects' as the outcome. The multilevel linear model had empty random-effects equations, meaning that the single-level linear model with no project team effects was a better fit for the data than the multilevel model. In other words, there was no nesting by the project team. Similarly, the multilevel Poisson model was not a better fit for the data than the single-level model with no project team effects. Therefore, I examined the extent to which network structure and spatial layout were associated with membership in prestigious and innovative project teams using Heckman sample selection regression models, and did sensitivity analysis using the multilevel mixed effects Poisson models. I ran the Heckman model to account for the fact that the theoretical framework (figure 1) assumes that in interacting and collaborating individuals identify expertise in others and form perceptions on the prestige of project teams through social cognition. Spatial cognition plays a role in how individuals navigate the physical space of the organization, which is related to the formation and maintenance of social ties and networks. Thus, the correlation between network structure and prestige outcomes is contingent on the association between spatial cognition and social networks. The Heckman sample selection model consists of two equations: a probit selection equation to model the likelihood that an individual will have a higher than median *degree* (centrality in the network based on number of ties), and, contingent on this, a linear outcome equation to model the number of prestigious and innovative projects that an individual is a member of.

The Heckman outcome equation was specified as follows. With ‘number of prestigious/innovative projects’ as the dependent variable, I fitted a model with ‘betweenness’ as the independent variable controlling for ‘gender’, ‘graduate degree’, and ‘manager’. For the Heckman selection equation, the dependent variable was ‘degree’ and the independent variable was ‘integration’.

The multilevel mixed effects Poisson model was specified similarly to both Heckman equations with ‘number of prestigious/innovative projects’ as the dependent variable, both social network measures (‘betweenness’ and ‘degree’) and spatial location (‘integration’) as the independent variables, and the ‘gender’, ‘graduate degree’, and ’manager‘ controls.

#### (ii) Group level

I examined the relation between network structure, spatial proximity, and the perceived prestige or innovativeness of a project team using linear regression. I ran a model with ‘size-weighted project prestige/innovativeness' as the dependent variable, and ‘interaction network, in-degree centralization’ and ‘spatial distance, average weighted degree’ as the independent variables.

### (d) Hypotheses

I examined the proposition that there is a correlation between social network structure, physical space or architecture, and (i) at the individual level, membership in prestigious projects, and (ii) at the group or team level, whether a project team is perceived as prestigious and innovative.

A person's location in the spatial system may impact whom they interact with on a regular basis, and the frequency of this interaction. The ‘integration’ variable captures the average depth of a space from all other spaces in the system. Individuals in spaces with higher integration will be more reachable by others in the organization compared with those in less integrated spaces. Similarly, individuals in more integrated spaces can access others in the organization more easily than those in more segregated spaces. Previous research has indicated that the greater the distance between two individuals, the lower the likelihood of a social relation between them [25]. Extrapolating from dyadic ties to relations among individuals in an organization, then, I would expect that a person who is on average a shorter depth or distance from others would have more social ties, which is captured by *degree* centrality. Recall that *degree* informs us that individuals with more ties or connections to others tend to be in favoured positions. I hypothesized that:

**H1**: Individuals with higher integration will have higher degree.

Social network centrality for individuals captures the extent to which they are in more or less favoured positions compared with others in the network. Being in a favoured position means that someone has fewer constraints and more opportunities compared with others. Network centrality identifies different ways in which individuals have unequal access to prestige or status in the organization. Specifically, ‘betweenness’ describes how individuals that are frequently on the shortest paths between others in the network are in favoured positions. This dimension of the *network* or *collective intelligence* in the organization should be correlated with the processes associated with individuals' selection into prestigious projects. I hypothesized that:

**H2**: Individuals with higher betweenness will have higher membership in prestigious projects.

The social network structure of a project team is a function of its individual members, their attributes, and the interactions among its members. There is mixed evidence for centralization and team outcomes. Some studies have found that centralization facilitates team performance [39] while others have found that the opposite is true [40]. Part of the reason for this discrepancy is that the teams studied have been in very divergent contexts with a focus on different types of outcomes, for example, student teams at a business school, professional soccer teams, etc. With respect to whether a team is perceived as prestigious and innovative, moderate to high levels of centralization are more likely to make a project team identifiable with one or a few key individuals that are high in prestige or status. This is likely to result in the team being perceived as prestigious relative to projects with a more equal distribution of connections, or lower centralization, which implies a lower likelihood of identification of the team with any one person regardless of their prestige or status. Specifically, I hypothesized that:

**H3**: Project teams with higher ‘interaction network, in-degree centralization’ are more likely to be perceived as prestigious.

Greater spatial proximity within a team would facilitate collaborative behaviours among its members [25], including the innovative collaborations [24], which should be correlated with a team's prestige perception. The team's ‘spatial distance, average weighted degree’ is a good indicator of the potential coordination and collaboration costs to a team as a result of how close or far apart team members are, taking into account the number of individuals in the team. For an individual in a team, in terms of spatial costs, walking to the workspaces for three other team members that are each located 10 feet away would be equivalent to walking to the workspace for one team member that is 30 feet away. Lower distances among team members, and hence lower *average weighted degree* should be correlated with greater ease of communication, coordination and collaboration (assuming face-to-face and not electronically mediated communication). Therefore, I hypothesized that:

**H4**: Project teams with a lower ‘spatial distance, average weighted degree’ are more likely to be perceived as prestigious.

## 3. Results

### (a) Individual level

With respect to the Heckman selection equation, ‘integration’ is positively and significantly correlated with ‘degree’, suggesting an association between spatial layout and social ties and networks. I cannot ascertain causality given the cross-sectional nature of the data. Nonetheless, the results align with the theoretical framework, which posits a link between physical space and social networks (figure 1). The results support hypothesis **H1**; there is a positive and significant correlation between ‘integration’ and ‘degree’. I did a sensitivity analysis (not shown) for the Heckman selection equation using single-level and multilevel logit regressions with the binarized ‘degree’ as the dependent variable, and single-level and multilevel linear regressions with raw *degree* as the dependent variable. The sensitivity analysis confirms the findings on **H1**. For example, the single-level linear model (model statistics: *F* = 5.91, *p* = 0.0208, *R*^2^ = 0.1560, *n* = 34) shows that a unit increase in ‘integration’ is associated with a roughly 69 unit increase in *degree* centrality (*p* = 0.021).

With respect to the Heckman outcome equation (‘number of prestigious/innovative projects’), social network position (‘betweenness’) is significantly correlated with the number of prestigious projects an individual is a member of, contingent on the relationship between spatial layout and number of social ties (selection equation). The results support hypothesis **H2**; individuals with higher ‘betweenness’ have higher membership in prestigious projects. I did a sensitivity analysis using multilevel mixed effects Poisson models (table 3), and the results support **H2**. Lastly, table 3 also reports the results of a likelihood ratio (LR) test which compares the multilevel model to a single-level model with no team effects (that is, a linear regression). The LR test statistic ‘chibar2(01)’ has a value of 0 with a *p*-value of 1, meaning that the single-level model offers a significantly better fit to the data than the multiple membership or multilevel model.

**Table 3.** Individual-level multilevel or multiple membership Poisson model for ‘number of prestigious/innovative projects’ (model statistics: Wald *χ*^2^ = 245.09, *p* = 0.0000, *n* = 264, groups = 30; LR test versus Poisson regression with no team effects, 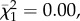 *p* = 1.0000).

predictor	estimate	s.e.	*p*-value
*intercept*	−*2.3865*	*0.3052*	*0.0000*
betweenness	0.0242	0.0094	0.0097
integration	5.7680	0.7279	0.0000
gender	−0.1520	0.0958	0.1127
graduate degree	0.4128	0.1224	0.0007
manager	0.9934	0.0966	0.0000

With respect to the controls, having a graduate degree and being a manager are both significantly correlated with the number of prestigious projects that an individual is a member of (table 4). However, there is no significant correlation between ‘gender’ and number of prestigious projects.

**Table 4.** Individual-level Heckman model for ‘number of prestigious/innovative projects’ (outcome equation) contingent on ‘degree’ (selection equation). Model statistics: Wald *χ*^2^ = 4.21 × 10^8^, *p* = 0.0000, *n* = 34.

predictor	estimate	s.e.	*p*-value
outcome equation: DV = ‘number of prestigious/innovative projects’			
*intercept*	*1.1139*	*0.3696*	*0.0026*
betweenness	0.0701	0.0334	0.0356
gender	−0.6041	0.4288	0.1589
graduate degree	1.2321	0.2004	0.0000
manager	2.5616	0.4014	0.0000
selection equation: DV = ‘degree’			
*intercept*	−*5.9466*	*1.2742*	*0.0000*
integration	13.8341	3.1944	0.0000

### (b) Group level

The ‘interaction network, in-degree centralization’ is positively and significantly correlated with ‘size-weighted project prestige/innovativeness’ (table 5). The ‘spatial distance, average weighted degree’ among the project members is negatively and significantly correlated with the ‘size-weighted project prestige/innovativeness’. The results support **H3**; there is a positive and significant correlation between ‘interaction network, in-degree centralization’ and a project team being perceived as prestigious. The results also support **H4**; there is a significant correlation between teams having a lower ‘spatial distance, average weighted degree’, and their perceived prestige (table 5).

**Table 5.** Group-level model output for ‘size-weighted project prestige/innovativeness’ (model statistics: *F* = 13.13, *p* = 0.0001, *R*^2^ = 0.4931, *n* = 30).

predictor	estimate	s.e.	*p*-value
*intercept*	*0.1711*	*0.0583*	*0.0067*
interaction network, in-degree centralization	2.4233	1.0655	0.0311
spatial distance, average weighted degree	−0.0001	0.0000	0.0004

## 4. Discussion

In this paper, I evaluated the individual- and group-level associations of *network collective intelligence* and physical space with prestige. I found that for individuals, as hypothesized, there is a significant correlation between physical space and social networks, and contingent on that, between network position and prestige. For project teams, I found that in accordance with my hypotheses there is a significant correlation between network structure and spatial proximity, and prestige.

For individuals, being located in a space that is easily accessible to and from all other spaces in the workplace is correlated with having more social ties in the organization. This finding is aligned with Zipf's Principle of Least Effort [41], which postulates that, all other factors being constant, people will choose the path of least resistance or effort. Therefore, if it is easier for people to find you physically, then they may be more likely to make the effort to contact you in the context of face-to-face interaction, and hence the significant correlation with the number of social ties that you may have. Previous work has established that spatial proximity is a greater determinant of social interaction than sociodemographic factors, such as common interests and family background [42]. Proximity has also been shown to amplify factors that are conducive to tie formation, including social similarity [43]. My results illustrate the importance of considering the spatial domain in examinations of how *network collective intelligence* is related to organizational outcomes.

I also found that, contingent on the social ties that you have, ‘betweenness’ network centrality—which captures a person's importance across the whole network—is positively and significantly correlated with the number of prestigious and innovative projects that the person is involved with. Individuals that are high in ‘betweenness’ are thought to have advantages with respect to obtaining novel information and other resources from disparate parts of the network. Previous studies have found a positive relationship between an individual's ‘betweenness’ and their perceived centrality by senior management [44]. Potentially, the increased visibility that is associated with individuals that have high ‘betweenness’ may explain not only why their expertise is more likely to be identified or recognized, but also why they are more likely to be placed in prestigious and innovative projects. The study findings suggest that an individual is more likely to be involved with prestigious projects when the focal person is frequently on the shortest paths between other pairs of individuals in the organization.

At the group level, ‘interaction network, in-degree centralization’ or the distribution of network ties is positively and significantly correlated with the team's prestige, suggesting that some centralization in a group may be useful. Recall that *centralization* is a network-level measure that captures how dispersed or concentrated individual centralities or positional advantages are in the network. Previous longitudinal research has established that moderate centralization facilitates team performance as evaluated by experts [39]. In a knowledge-based workplace, there may be no clear-cut path to desirable organizational outcomes. Thus, there may be a level of uncertainty about the performances of others, and even technological uncertainty [45]. This uncertainty may make a person that is high in prestige or status a desirable collaboration partner as others in the organization are more likely to infer expected performance from perceived prestige or status [45]. Equally or even more likely, project teams may be formed around individuals with high levels of expertise (and hence high prestige) in the subject matter that is pertinent to the project. These individuals may either select or attract other individuals who may not have similarly high subject matter expertise (hence low prestige) but who may have other complementary skills that are essential to the success of the team. I do not have the data to ascertain which mechanism drives the process that results in the significant correlation between ‘interaction network, in-degree centralization’ and a team's perceived prestige. However, it is logical to conclude that pairing high-prestige individuals to others with lower prestige would lead to project teams with high ‘interaction network, in-degree centralization’. The association of the team with the high prestige individual may very well confer prestige and status to the individual's team in a climate of uncertainty about organizational outcomes.

The ‘spatial distance, average weighted degree’ is negatively and significantly correlated with a project team's prestige and innovativeness. Specifically, teams with a lower *average weighted degree* are perceived as more prestigious and innovative. Simply put, co-location and distance matter [46]. Not only may teams with a lower ‘spatial distance, average weighted degree’ provide more opportunities for the encounters that enhance collaborative behaviours, but the reduced spatial costs of face-to-face communications among team members may correlate to higher impact outputs [47]. After all, there are characteristics of face-to-face interactions that are irreplaceable by technology-mediated communication [46]. In the space–time contexts of these interactions in the organizational workplace, it would be advantageous for teams to have smaller physical distances—the ‘spatial distance, average weighted degree’—among team members.

## 5. Limitations and conclusion

A key limitation is that this was a cross-sectional study meaning that while I could identify novel correlations between *network collective intelligence* and spatial layout, and prestige, I could not ascribe causality or identify causal mechanisms for these correlations. Future studies could build on my findings by using longitudinal data to examine dynamics of *network collective intelligence* and physical space, and the temporal relationships between these two factors and organizational outcomes such as prestige.

This study examined the individual- and group-level effects of *network collective intelligence* and physical space on prestige. Network position and physical space have significant correlations with the number of prestigious projects an individual is a member of, contingent on the association between social networks and physical space. Furthermore, network structure and physical space are correlated with the perceived prestige and innovativeness of a project team. These findings on *network collective intelligence* and physical space contribute to the understanding of individual- and team-level correlations between social networks and organizational outcomes in physical space. Future studies could extend this work by incorporating longitudinal locational data that allow for causal linkages from movement in physical space by individuals to the face-to-face encounters and interactions that are essential to the formation and maintenance of social ties. The increased availability of location-tracking technology has significantly decreased the costs of collecting these types of data, making the collection and analysis of location data a fitting way to extend the findings in this study.

## Supplementary Material

Group Level Data 04112018.dta

## Supplementary Material

Group Level Code 04112018.do

## Supplementary Material

Individual Level Data 04112018.dta

## Supplementary Material

Individual Level Code 04112018.do

## Supplementary Material

Individual Level Project-Panel Data 04112018.dta

## Data accessibility

Datasets and code are provided as the electronic supplementary material.
